# Are restrictive guidelines for added sugars science based?

**DOI:** 10.1186/s12937-015-0114-0

**Published:** 2015-12-12

**Authors:** Jennifer Erickson, Joanne Slavin

**Affiliations:** Food Science and Nutrition Department, University of Minnesota, 1334 Eckles Ave, St Paul, MN 55108 USA

**Keywords:** Added sugar, Free sugar, Sugar recommendations, Nutrition facts labels, Dietary Guidelines

## Abstract

Added sugar regulations and recommendations have been proposed by policy makers around the world. With no universal definition, limited access to added sugar values in food products and no analytical difference from intrinsic sugars, added sugar recommendations present a unique challenge. Average added sugar intake by American adults is approximately 13 % of total energy intake, and recommendations have been made as low 5 % of total energy intake. In addition to public health recommendations, the Food and Drug Administration has proposed the inclusion of added sugar data to the Nutrition and Supplemental Facts Panel. The adoption of such regulations would have implications for both consumers as well as the food industry. There are certainly advantages to including added sugar data to the Nutrition Facts Panel; however, consumer research does not consistently show the addition of this information to improve consumer knowledge. With excess calorie consumption resulting in weight gain and increased risk of obesity and obesity related co-morbidities, added sugar consumption should be minimized. However, there is currently no evidence stating that added sugar is more harmful than excess calories from any other food source. The addition of restrictive added sugar recommendations may not be the most effective intervention in the treatment and prevention of obesity and other health concerns.

## Introduction

Governments and health organizations worldwide have published dietary guidance for sugar intake [1]. Despite access to the same published literature, recommendations vary greatly and create confusion for health practitioners and consumers. Since 1980, Dietary Guidelines for Americans (DGA) has recommended we “avoid too much sugar”, yet dietary advice has typically recommended foods high in sugar, such as fruits and dairy products. As a way to clarify the types of sugar to avoid, the terms added sugars and free sugars are used. Added sugar recommendations have been in existence since 2002, with recent recommendations becoming progressively more restrictive over the years [1]. This paper addresses current and proposed added sugar recommendations and assesses their practicality within the United States.

## Definition of added sugars

No universally accepted definition for added sugars exist (Table 1). The Food and Drug Administration (FDA) classifies added sugars as, mono and disaccharides added to foods during production including sugars, syrups, fruit juice concentrates, honey, etc. This would not include sugars that naturally exist in foods, such as sugars in fruits or dairy products [2]. A common point of contention between institutions is whether or not fruit juice should be included as added sugars. The proposed revisions to the Nutrition Facts and Supplements Label published by the FDA in 2014 classifies fruit juice concentrate added to food products as added sugar, while juice not from concentrate as not added sugar. In comparison, the USDA recommendations do not specify that fruit juice from concentrate contributes to added sugar totals [2, 3].

**Table 1:** Added sugar definitions and distinctions from various agencies

Food and Drug Agency (FDA) Proposed Nutrition Facts Label [3]	Mono- and disaccharides added to foods during production including: sugars, syrups, fruit juice concentrates, honey, etc.
United States Department of Agriculture (USDA) Choose MyPlate [2]	Sugars added during processing and preparation. Includes: sugars, syrups, honey, nectars, etc.
	Excludes: fruit juice and fruit juice concentrates
World Health Organization (WHO) Free Sugar Guidelines [4]	Mono- and disaccharides added to foods during processing or by the consumer during preparation. Includes: Sugars, syrups, honey, fruit juice and fruit juice from concentrate.
Scientific Report of the 2015 Dietary Guidelines Advisory Committee [8]	Sugars, syrups, isolated naturally occurring sugars (ex: fruit juice concentrate) and other caloric sweeteners

In addition to various definitions for the term “added sugars”, the World Health Organization (WHO) utilizes the term “free sugar”. Free sugar is similar to added sugars, as it includes all sugars and syrups added to foods; however, free sugar also includes sugars naturally present in fruit juices and fruit juice concentrates [4]. Free sugar includes sugars naturally found in fruit juice that is consumed as a beverage as well as fruit juices added to food products. Assessing added sugar intake and compliance with recommendations would be extremely difficult without a clear and established definition of the term “added sugar” and, specifically, how fruit juice should contribute to added sugar values.

## Function of added sugar

Added sugars are chemically identical to sugar that naturally occurs in food products [1]. The body cannot distinguish the source of the nutrient and processes the sugar in the same way. Sugar may be added to food products for many reasons, the most obvious reason being adding sweetness and enhancing the palatability of foods. Although this function of sugar is often opposed and criticized, many American consumers would not find a number of “healthy” foods palatable without added sugar. Some examples include cranberries, yogurt and oatmeal. Nutrition professionals often encourage clients to consume these foods as part of a healthy diet, even with some added sugar [5].

Another function of sugar within food products is texture enhancement. Sugar produces a tender texture in baked products, and inhibits ice crystallization in frozen products. Sugar provides body to products and, when removed, has to be substituted with bulking agents to achieve a similar mouth feel [6]. Carmelization and maillard browning are both reactions specific to sugar and provide an appearance expected in food products. Sugar also plays a role in food safety by inhibiting the growth of microorganisms at high concentrations. By binding with water molecules, sugar can maintain moisture contents in products lengthening the shelf life [6]. Overall, it is important to remember that sugar functions in many capacities beyond just flavor.

## Added sugar intake in the American diet

Added sugar intake is on average 13 % of total energy intake in adults and 16 % in children, consistently decreasing with age [7]. Added sugar consumption has declined in all age groups from NHANES data taken in 2001–2004 to data from 2007–2010. Meanwhile, rates of obesity did not mimic the decline over the same time period [8]. According to NHANES data from 2009–2010, 47 % added sugars in the American diet come from beverages, 31 % from snacks and sweets, 8 % from grains, and 14 % from the categories of dairy, mixed dishes, condiments, fruits and fruit juice and vegetables combined [8]. While there is room for improvement in the American diet, this decrease in added sugar intake is encouraging and understanding the main sources of added sugars provides a direction to focus our efforts.

## Added sugar recommendations in America

In 2002, the Institute of Medicine (IOM) Dietary Reference Intakes recommended that less than 25 % of total energy should come from added sugars. The recommendation is based on the concept that foods containing high amounts of added sugars are typically high in calories and low in micronutrients [9]. The idea that added sugars are “empty calories” is a commonly cited reason that added sugar recommendations are necessary. Diets containing a large amount of energy as “empty calories” can lead to micronutrient malnutrition or over consumption of calories. Consuming the daily recommendation of all nutrients within an individual’s estimated energy requirement is challenging when the individual is consuming a large portion of his or her calories as empty calories. Repeated consumption of empty calories without compensation from other nutrients can lead to weight gain.

The current 2010 Dietary Guidelines for Americans, includes solid fats and added sugars (SoFAS) in their recommendation of 5–15 % of total energy from solid fats and added sugars [10]. Minimizing SoFAS consumption is encouraged to reduce excess calorie consumption and to replace foods high in added sugars with foods lower in added sugars and greater nutrient density. SoFAS consumption above the recommendation is considered to be incompatible with the USDA Food Patterns, likely exceeding calorie limits or obtaining inadequate micronutrient intake [10].

The USDA Food Patterns were created to assist the public in following Dietary Guideline recommendations, providing amounts of food from each food group to achieve optimal nutrient intake [11]. The USDA Food Patterns groups added sugars and solid fats together and recommends adult females and adult males to limit “empty calorie” intake to 120–250 calories per day and 160–330 calories per day, respectively, depending on caloric needs [8]. Consumption of empty calories is typically above the current recommendations in all age groups; almost 90 % of Americans exceed the USDA food pattern recommendations [8]. The evolution of the concept of discretionary calories (2005 DGAs) to empty calories (2010 DGAs) is explained by Nicklas and O’Neil [12]. The authors also explain that the reduction of solid fats and added sugars is to remove calories from the diet, not because solid fats and added sugars are linked to negative health outcomes [12].

The World Health Organization not only cites the effects of excess calories, but also the impact that sugar can have on dental health. The current World Health Organization recommendation of fewer than 10 % of total calories from free sugars was set in 2003 [13]. However in 2015, WHO set a conditional recommendation suggesting that less than 5 % of total energy should come from free sugars [4]. This conditional recommendation proposed by WHO is based on a positive association between free sugar intake and dental caries among children [4]. Sugar consumption has been positively associated with risk of dental disease. According to a meta-analysis published in 2014, there is moderate evidence indicating that a free sugar intake less than 10 % of total calories was associated with decreased risk of dental caries [14]. Further decrease in caries was seen in Japanese surveys, taken between 1959 and 1960, when free sugar intake approached 5 % of total calories [15]. The area surveyed had low fluoride exposure so it may not be an accurate model to extrapolate to areas with good fluoride exposure in the United States. Although, the WHO states that all populations, regardless of fluoridation, could possibly see improvement in dental caries with decreased free sugar intake [4, 15]. Additionally, the sugar consumption data was calculated by looking at sugar consumption per capita, added sugar intake compared to incidence of dental carries for each individual was not known [15]. The limitations of the Japanese studies prevented the WHO from setting a strong recommendation to consume fewer than 5 % of calories from free sugars [4]. However, because dental caries occur throughout the lifespan, consuming fewer free sugars is estimated to have a cumulative effect and result in decreased dental problems later in life and no evidence of harm was seen in diets containing fewer than 5 % energy from free sugars [4].

## Dietary Guidelines for Americans 2015

The release of the Scientific Report of the 2015 Dietary Guidelines Advisory Committee (DGAC) in February 2015 brought further attention to added sugars. The DGAC report placed a large focus on added sugars, making it one of the five “cross-cutting topics” [8]. The Committee reexamined the evidence surrounding the potential health effects of added sugars. The DGAC assessed the evidence that added sugar negatively impacts the health risks for obesity, type II diabetes, cardiovascular disease and dental carries. The DGAC determined, based on the available evidence, there was a strong correlation between added sugars and negative health risks. Most of the cited evidence examines the association between sugar sweetened beverage (SSB) consumption and the health risk rather than the consumption of added sugar from variety of foods [8]. It is easier to count consumption of SSBS with food frequency instruments used in epidemiologic studies than to estimate total added sugar intake since few databases included information on added sugars. While SSB consumption may be the best method available for added sugar estimates, it is not without its limitations including possible confounding variables within the population. According to a recent study of over 12,000 participants, individuals reporting to consume one or more SSB per day were significantly more likely to smoke, consume fewer fruits and vegetables and report a sedentary lifestyle [16]. No discussion of if these confounding variables were considered in the DGAC report [8].

After an examination of the evidence and diet modeling, the DGAC suggested an appropriate intake of calories from added sugars to be between 4–6 % and set a maximal intake of 10 % total energy from added sugars [8]. After a period of time allowing for comments from the general public, the USDA and Department of Health and Human Services will assess evidence behind the recommendation from the USDA to set the Dietary Guidelines for Americans 2015 added sugar recommendation [8].

With this suggested restriction on added sugars, the DGAC recognizes that the logical consequence of removing added sugars from the diet and food products would be replacing the added sugars with low calorie sweeteners. However, the DGAC report advises against this replacement due to the minimal evidence regarding long-term effect of low calorie sweeteners. Instead, the DGAC encourages the replacement of sugar-sweetened beverages with water and does not suggest a replacement in food products. Removal of sugar from products will change the taste, texture and shelf-life of products due to the functions of sugar previously discussed [6]. The sugar must be replaced with other ingredients and, if not low calorie sweeteners, what would be a better alternative? Evidence exists to support the use of low-calorie sweeteners in weight reduction [8] and many consumers utilize this approach to support weight loss. The FDA recognizes artificial and low-calorie sweeteners as safe for consumption, and the Academy of Nutrition and Dietetics advises that non-nutritive sweeteners can fit into a healthy diet [17]. Identifying alternative sweeteners or ingredients to produce comparable food and beverage products is essential in changing the consumption patterns in Americans. Taste is consistently the most important buying factor for most Americans, and without great tasting alternatives consumers are not likely to make dietary changes [18].

## Proposed addition of “Added sugar” to nutrition facts panel

Currently, there is no easy way for consumers, researchers or health professionals to track added sugar consumption and assess compliance with recommendations. Very few databases exist that calculate added sugars, and, due to the various added sugar definitions, the information obtained from these databases may result in a range of added sugar values. In March 2014, the FDA proposed changes to the Nutrition Facts Panels to assist consumers in making more educated food choices that would lead to a healthy diet consistent with Dietary Guidelines for Americans. The recent proposal to update the Nutrition Facts Panel advocates for the addition of an “Added sugars” category below the “Sugars” category, that would provide a way to track and compare added sugars [19]. The proposed amendments to the food labels suggest displaying added sugar in grams. The DGAC report supports such changes to the food labels and recommends displaying added sugar values in grams, teaspoons and percent daily value [8].

A supplemental proposed rule regarding the Nutrition Facts Panel was published in July of 2015. The FDA proposed to establish a less than 10 % Daily Reference Value (DRV) and to include the percent Daily Value (DV) on the Nutrition Facts Panel [20]. The supplemental proposed rule cites the 2015 DGAC report as their basis for instituting an added sugar DRV. The proposed rule states that the 2015 DGAC showed a “strong association between a dietary pattern of intake characterized, in part, by a reduced intake of added sugars and a reduced risk of cardiovascular disease” [20]. Traditionally, DRVs and %DVs have been established for nutrients where an average dietary requirement can be determined from available scientific evidence [21]. The data used to determine the <10 % DRV for added sugar was based primarily on diet modeling conducted for the 2015 DGAC. No DRV is has been proposed for total sugars at this time due to lack of available evidence for a reference intake [8, 20].

The purpose of the FDA’s changes to the Nutrition Facts Panel is to help consumers make choices leading to healthier diets, however; the addition of the “added sugar” category may not provide much novel knowledge to consumers. According to NHANES 2009–2010, nearly 80 % of added sugars come from sugar-sweetened beverages (47 %) and snacks and sweets (31 %) [8]. The proposed changes to the food labels would require food companies to invest their resources to calculate the added sugar in their products, when the majority of added sugar consumed comes from obvious sources of sugar. Just over 20 % of added sugars consumed by Americans come from non-obvious forms where the consumer would benefit from the knowledge of added sugars on the food labels, if they choose to read the label [8].

A study presented by the International Food and Information Council showed that the addition of the category “Added Sugars” to the Nutrition Facts Panel reduced the consumer comprehension of the food label. The percent of participants able to accurately identify the total grams of sugar dropped from 92 to 55 % when the added sugars category was included, with more than half the participants adding added sugars with the sugar category [22]. A similar study was later conducted by the FDA, finding consistent results. Ability to accurately identify the grams of sugar per serving decreased from 81 % to 65 % when the label was updated to the proposed format [23].

Other research supports that consumers are interested in added sugar labeling. Kyle & Thomas report that consumers believe Nutrition Facts labeling for added sugar will be more helpful than confusing [24]. A study in European Union found that consumers expect that a reduction in free sugars in a product will be linked to a reduction in the calorie content of the food [25]. Nevertheless, a consumer study with cereals found that participants rated cereals containing “fruit sugar” as healthier than cereals containing “sugar”, although there were no differences in nutrient content between the cereals [26]. Total sugar analysis is challenging enough. When sugar content of commercial foods targeted to infants and children was conducted by a blinded laboratory analysis of accepted chemical methods, nutrient label data underestimated or overestimated actual sugar content routinely. The authors suggest that more effort should be made to standardize methods for sugar labeling of foods, especially foods targeted to children [27].

Health Canada recently removed the added sugars category from their proposed nutrition facts table and included a 20 % DV for total sugar [28]. Consumer research by the Canadian government found that information about carbohydrates and total sugars was confusing when the table included added sugars. It also found the % DV approach to be useful and easy to understand. They state: “*the proposal to declare the amount of added sugars was popular among consumers and health stakeholders (including health professionals). However, industry stakeholders questioned the scientific basis of requiring the declaration of added sugar given that the body metabolizes naturally occurring and added sugars in the same way. Similarly, the inability of analytical method to distinguish between naturally occurring and added sugars would contribute to significant compliance and enforcement challenges.”*

Because added sugars are not chemically different from intrinsic sugars, there is no way to analytically determine the amount of added sugar in a food product [1]. Food manufacturers would have to calculate the added sugars based on the recipe in order to determine the added sugar content in each product every time the product is reformulated. Without a clear definition of added sugars the resultant labeling will likely be inconsistent. The FDA would require food companies to document, maintain and provide records on product composition to verify the published value of added sugars [3]. Due to competition within the food industry and the proprietary nature of the formulations, food manufacturers would be very resistant to release such information. Moreover, each of these steps will require additional time, money and an acquired skill set that smaller food companies may not have the resources to comply with.

## Conclusion

Excess calorie consumption can lead to weight gain and increased risk of obesity and obesity-related co-morbidities [8]. Empty calories which include solid fats and added sugars play a role in this when consumed in abundance. Added sugars are low in nutrient density and calories from added sugars can add up quickly if the individual is not conscious of their diet. However, there is no evidence suggesting that excess calories from added sugars specifically are worse than excess calories from any other food source. Much of the evidence linking added sugars to chronic disease is done measuring sugar sweetened beverages rather than percent calories from all added sugars [8]. With nearly half of added sugar consumption in America being attributed to sweetened beverages, perhaps encouraging healthy beverage alternatives to sugar sweetened beverages should be the focus, rather than zeroing in on all added sugars.

Regulatory attempts to tax sugar sweetened beverages in countries, such as Mexico and communities in the US, including Berkeley, CA may increase tax revenue, but whether these more aggressive approaches can limit calorie intake and/or improve health outcomes await clinical trial results.

Recommendations as low as 5 % total energy from free sugars are likely too restrictive for most Americans to achieve [29]. Added sugars should be consumed at a minimum as they are often a source for surplus calories in the American diet; however, stringent recommendations and mandatory food labeling are likely not the most effective ways to reduce added sugar and excess calorie consumption. Education on healthy beverages, snack choices and portion sizes may be a better starting point for reducing empty calorie intake.

## Abbreviations

DGA: Dietary Guidelines for Americans
DGAC: Dietary Guidelines Advisory Committee
DRV: Daily reference value
DV: daily value
FDA: Food and Drug Administration
IOM: Institute of Medicine
SoFAS: solid fats and added sugars
SSB: sugar sweetened beverage
WHO: World Health Organization
